# COVID-19 Outbreak Among Employees at a Meat Processing Facility — South Dakota, March–April 2020

**DOI:** 10.15585/mmwr.mm6931a2

**Published:** 2020-08-07

**Authors:** Jonathan Steinberg, Erin D. Kennedy, Colin Basler, Michael P. Grant, Jesica R. Jacobs, Dustin Ortbahn, John Osburn, Sharon Saydah, Suzanne Tomasi, Joshua L. Clayton

**Affiliations:** ^1^CDC COVID-19 Response Team; ^2^Epidemic Intelligence Service, CDC; ^3^South Dakota Department of Health; ^4^Laboratory Leadership Service, CDC.

On March 24, 2020, the South Dakota Department of Health (SDDOH) was notified of a case of coronavirus disease 2019 (COVID-19) in an employee at a meat processing facility (facility A) and initiated an investigation to isolate the employee and identify and quarantine contacts. On April 2, when 19 cases had been confirmed among facility A employees, enhanced testing for SARS-CoV-2, the virus that causes COVID-19, was implemented, so that any employee with a COVID-19–compatible sign or symptom (e.g., fever, cough, or shortness of breath) could receive a test from a local health care facility. By April 11, 369 COVID-19 cases had been confirmed among facility A employees; on April 12, facility A began a phased closure* and did not reopen during the period of investigation (March 16−April 25, 2020). At the request of SDDOH, a CDC team arrived on April 15 to assist with the investigation. During March 16–April 25, a total of 929 (25.6%) laboratory-confirmed COVID-19 cases were diagnosed among 3,635 facility A employees. At the outbreak’s peak, an average of 67 cases per day occurred. An additional 210 (8.7%) cases were identified among 2,403 contacts of employees with diagnosed COVID-19. Overall, 48 COVID-19 patients were hospitalized, including 39 employees and nine contacts. Two employees died; no contacts died. Attack rates were highest among department-groups where employees tended to work in proximity (i.e., <6 feet [2 meters]) to one another on the production line. Cases among employees and their contacts declined to approximately 10 per day within 7 days of facility closure. SARS-CoV-2 can spread rapidly in meat processing facilities because of the close proximity of workstations and prolonged contact between employees (*1*,*2*). Facilities can reduce this risk by implementing a robust mitigation program, including engineering and administrative controls, consistent with published guidelines (*1*).

## Investigation and Findings

Facility A, which employed 3,635 persons in 38 departments, harvests and processes animals during two shifts per day. A third shift sanitizes the facility. On March 24, SDDOH was notified that an employee had received a positive SARS-CoV-2 test result; SDDOH began an investigation that day. The employee worked in department A during the first shift. He had last worked on March 14, developed symptoms on March 16, and was tested on March 22. On March 19, a first-shift employee in department B became ill. The following day, two additional first-shift department A employees and one second-shift department C employee developed symptoms. On March 21, one first-shift department B employee developed symptoms, for a total of six COVID-19 cases among employees. During March 22–28, 18 employees from department B developed COVID-19 symptoms, resulting in the department’s temporary closure on April 3; 15 cases in employees from nine other departments also occurred that week. On April 3, facility A also began screening all employees for fever, installing physical barriers on the production line, and amending the employee dress code to include optional masks, which were required as of April 13, 1 day after the phased closure of facility A began. By April 4, a total of 247 employees from 23 departments had developed COVID-19.

A COVID-19 case was defined as a positive SARS-CoV-2 reverse transcription–polymerase chain reaction test result in a person who had onset of COVID-19–compatible symptoms, or who was tested in the absence of symptoms, before April 26 (i.e., 14 days after phased closure began). Illness onset date was defined as the date COVID-19–compatible symptoms first appeared (or the specimen collection date, if no symptom onset date was documented). All reported cases were investigated by SDDOH to determine patient symptom onset date, identify and trace contacts, and describe patients’ clinical course of illness. Lists with employee characteristics provided by facility A were used, along with SDDOH case investigation data, to identify cases associated with facility A and to calculate attack rates. Employees’ contacts were identified through interviews conducted by SDDOH and were defined as persons who were within 6 feet (2 meters) of an employee who had a positive SARS-CoV-2 test result for at least 5 minutes during the employee’s infectious period (i.e., from symptom onset to discontinuation of isolation). On April 1, the infectious period was expanded to include persons who had contact with persons with known COVID-19 during the 48 hours before symptom onset, in accordance with changing CDC guidance. Employees who did not work during March 2–April 25 were excluded from analysis. Departments were aggregated into seven department-groups as determined by the facility’s supervisory structure: Bacon, Conversion,^†^ Cut, Harvest, Sausage, Smoke meat, and Other. Department-groups tended to consist of departments that performed similar functions under similar conditions and received COVID-19-related guidance and communication through similar channels. Attack rates were calculated by shift, department-group, and compensation status. A community resident was defined as a resident of one of the two counties that compose the city where facility A is located who was neither an employee of facility A nor a known contact of a facility A employee. SAS (version 9.4; SAS Institute) was used to conduct statistical analyses. This investigation was determined by CDC to be public health surveillance.^§^

During March 16–April 25, among 3,635 facility A employees, 929 (25.6%) met the COVID-19 case definition, including 895 (96.3%) who were symptomatic (Table 1) (Figure). During this period, facility A employees represented 920 (41.8%) of the 2,199 COVID-19 cases identified among community residents. Among 2,403 identified employee contacts, 210 (8.7%) had confirmed COVID-19 (illness onset range = March 30–April 25). The median employee age was 42 years (range = 18–81 years), and the median employee contact age was 29 years (range = 0–85 years). Among employees diagnosed with COVID-19, 34 (3.7%) were asymptomatic, as were six (2.9%) contacts and 53 (4.9%) community residents. Among those with symptoms, symptom onset date was not documented for 33 (3.7%) employees, 10 (4.9%) contacts, and 28 (2.7%) community residents. The earliest symptom onset date reported among community residents with diagnosed COVID-19 was February 24.

**TABLE 1:** Demographic* and clinical characteristics of COVID-19 patients among employees at a meat processing facility, their contacts, and community residents^†^ — South Dakota, February 24−April 25, 2020

Characteristic	No. (%)		
	Employees (n = 929)	Contacts (n = 210)	Community residents (n = 1,086)
**Demographic**			
**Sex**			
Female	333 (35.8)	124 (59.1)	592 (54.5)
Male	596 (64.2)	86 (41.0)	494 (45.5)
**Age, yrs, median (range)**	42.0 (18–81)	29.0 (0–85)	40.0 (0–100)
**Age group (yrs)**			
<18	0 (—)	37 (17.6)	74 (6.8)
18–44	512 (55.1)	111 (52.9)	579 (53.3)
45–54	235 (25.3)	28 (13.3)	158 (14.6)
55–64	156 (16.8)	24 (11.4)	155 (14.3)
≥65	26 (2.8)	10 (4.8)	120 (11.1)
**Clinical**			
Symptomatic	895 (96.3)	204 (97.1)	1033 (95.1)
Hospitalized	39 (4.2)	9 (4.3)	130 (12.0)
ICU admission	14 (1.5)	3 (1.4)	22 (2.0)
Died	2 (0.2)	0 (—)	25 (2.3)

**Abbreviations:** COVID-19 = coronavirus disease 2019; ICU = intensive care unit.

* Race and ethnicity data were incomplete and are not presented.

^†^ A resident of one of the two counties that compose the city where facility A is located who was neither an employee of facility A nor a contact of a facility A employee.

**FIGURE:**
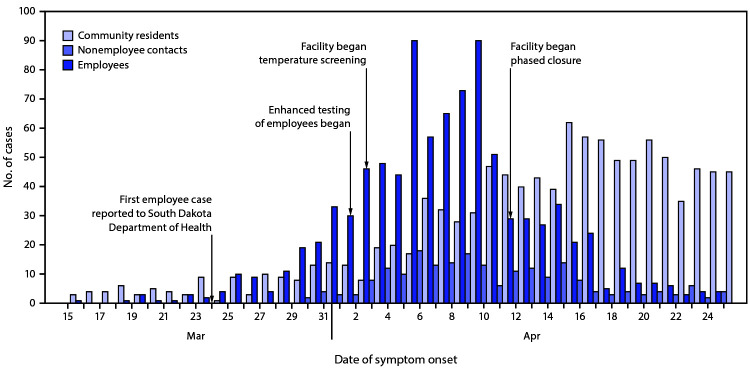
Confirmed COVID-19 cases among employees at a meat processing facility (n = 929), their contacts (n = 210), and community residents* (n = 1,086) and facility mitigation strategies,^†^ by date of illness onset^§^^,^^¶^ (N = 2,225) — South Dakota, February 24−April 25, 2020 **Abbreviation:** COVID-19 = coronavirus disease 2019. * A person who resided in one of the two counties that make up the city in which facility A is located who was neither an employee of facility A nor a contact of an employee. ^†^ Beginning April 12, the facility did not slaughter any more animals. During April 12–14, the facility processed animals that had already been slaughtered, shipped finished product, and progressively closed departments. From April 15 onward, only staff members necessary for maintenance, cleaning, and sanitization of the facility, transportation of remaining product, and implementation of COVID-19 prevention activities reported to work. **^§^** The date COVID-19–compatible symptoms first appeared or, if no symptom onset date was documented during the investigation, specimen collection date. For asymptomatic persons, SARS-CoV-2 specimen collection date is reported. ^¶^ During February 24–March 14, 11 community residents had COVID-19 illness onset.

Among employees with COVID-19, 39 (4.2%) were hospitalized; the median age of hospitalized patients was 60 years (range = 28–73 years). As of June 14, 11 hospitalized patients had been discharged after a median length of stay of 6.5 days (range = 1–69 days). Nine (4.3%) contacts who developed COVID-19 were hospitalized; the median age of hospitalized contacts was 64 years (range = 23–79 years), and they were hospitalized for a median of 10 days (range = 1–15 days). As of June 14, two employees with COVID-19 had died.

The attack rate at facility A during March 16–April 25 was 25.6% (Table 2). The highest attack rates occurred in the Cut (30.2%), Conversion (30.1%), and Harvest (29.4%) department-groups. The first, second, and third shifts had similar attack rates. The attack rate among nonsalaried employees was 26.8% and among salaried employees was 14.8%. During the first 3 weeks of the outbreak, the overall attack rate increased approximately fivefold per week (week 1 = 0.2%, week 2 = 1.2%, and week 3 = 6.8%). During the fourth week of the outbreak, an average of 67 employee COVID-19 cases were occurring per day. Within 7 days of facility closure, cases among employees declined to approximately 10 per day.

**TABLE 2:** COVID-19 cumulative attack rates among employees (N = 3,635) at a meat processing facility, by week of illness onset* — South Dakota, March 15−April 25, 2020

Employee division (no. with available information)	COVID-19 cases, no. (%)					
	March 15−21	March 15−28	March 15− April 4	March 15− April 11	March 15− April 18	March 15− April 25
**Compensation**						
Nonsalaried (3,372)	6 (0.2)	39 (1.2)	240 (7.1)	691 (20.5)	848 (25.1)	890 (26.4)
Salaried (263)	0 (0.0)	0 (0.0)	7 (2.7)	26 (9.9)	38 (14.4)	39 (14.8)
**Total (3,635)**	**6 (0.2)**	**39 (1.2)**	**247 (6.8)**	**717 (19.7)**	**886 (24.4)**	**929 (25.6)**
**Department-group**						
Cut (882)	0 (0.0)	4 (0.5)	64 (7.3)	211 (23.9)	251 (28.5)	266 (30.2)
Conversion^†^ (575)	5 (0.9)	24 (4.2)	91 (15.8)	154 (26.8)	170 (29.6)	173 (30.1)
Harvest (428)	0 (0.0)	2 (0.5)	26 (6.1)	88 (20.6)	121 (28.3)	126 (29.4)
Smoke meat (357)	0 (0.0)	1 (0.3)	14 (3.9)	66 (18.5)	83 (23.2)	86 (24.1)
Bacon (234)	1 (0.4)	2 (0.9)	10 (4.3)	42 (17.9)	52 (22.2)	54 (23.1)
Sausage (151)	0 (0.0)	1 (0.7)	3 (2.0)	23 (15.2)	32 (21.2)	33 (21.9)
Other (745)	0 (0.0)	5 (0.7)	32 (4.4)	107 (14.4)	139 (18.7)	152 (20.4)
**Total (3,372)**	**6 (0.2)**	**39 (1.2)**	**240 (7.1)**	**691 (20.5)**	**848 (25.1)**	**890 (26.4)**
**Shift**						
First (1,744)	5 (0.3)	32 (1.8)	142 (8.1)	381 (21.8)	463 (26.5)	485 (27.8)
Second (1,459)	1 (0.1)	6 (0.4)	93 (6.4)	278 (19.1)	347 (23.8)	359 (24.6)
Third (167)	0 (0.0)	0 (0.0)	4 (2.4)	30 (18.0)	36 (21.6)	44 (26.3)
**Total (3,370)**	**6 (0.2)**	**38 (1.1)**	**239 (7.2)**	**691 (20.5)**	**846 (25.1)**	**888 (26.4)**

**Abbreviation:** COVID-19 = coronavirus disease 2019.

* COVID-19 illness onset was defined as the date COVID-19–compatible symptoms first appeared or, if no symptom onset date was documented during the investigation, specimen collection date. For asymptomatic cases, SARS-CoV-2 specimen collection date is reported.

^†^ The process of further refining initial cuts of meat into finished fresh meat products.

## Public Health Response

Beginning March 24, SDDOH investigated all cases among facility A employees and their contacts. Persons with confirmed COVID-19 were instructed to self-isolate. Contacts of patients were traced, instructed to quarantine, and actively monitored for signs and symptoms of COVID-19 using CDC’s Text Illness Monitoring (TIM) system.^¶^ Contacts who developed symptoms of COVID-19 were counseled and referred to a health care provider to be evaluated for SARS-CoV-2 testing.

## Discussion

Outbreaks of COVID-19 have been described among employees in congregate settings (*3*–*5*). This large outbreak of COVID-19 among employees at a meat processing facility highlights the potential for rapid transmission of SARS-CoV-2 in these types of facilities. Factors that might have contributed to infection among employees at this facility include high employee density in work and common areas, prolonged close contact between employees over the course of a shift, and substantial SARS-CoV-2 transmission in the surrounding community (*6*).

The Cut, Conversion, and Harvest department-groups, in which numerous employees tended to work <6 feet (2 meters) from one another on the production line, experienced the highest attack rates. Salaried employees, who typically had workstations that could be adjusted to maintain distancing and did not work in close proximity to other employees on the production line, had a lower attack rate than did nonsalaried employees. These differences highlight the importance of engineering controls (e.g., physical barriers) and administrative controls (e.g., cohorting employees) in mitigating the risk for SARS-CoV-2 transmission in meat processing facilities (*1*). Consistent and correct use of masks can also prevent presymptomatic or asymptomatic employees with SARS-CoV-2 infection from transmitting the virus to others (*1*).

Although cases were confined to three departments during the first week of the outbreak, the number of affected departments increased rapidly. Contact between employees in common areas (e.g., cafeterias, locker rooms, and equipment-dispensing locations) might have facilitated spread among employees in different departments. Visual cues to maintain physical distancing and staggered shifts and break times might reduce risk for transmission among employees in these areas (*1*). Transmission among employees who work in different departments might have also occurred outside the facility (e.g., carpooling, cohabitating, and socializing outside work).

Employees working the first, second, and third shifts experienced similar attack rates, although employee density in the facility is lowest during the third shift, and sanitizing duties entail physical distancing and the use of personal protective equipment. Transmission among third shift employees might have occurred in common areas or outside the facility.

Although COVID-19 cases among employees declined to approximately 10 cases per day within 7 days of facility closure, some decrease was observed before closure. Implementation of control measures before closure of facility A might have contributed to this decrease. Employee testing decreased after facility closure, which also might have contributed to the apparent reduction in cases.

The findings in this report are subject to at least five limitations. First, during a period of limited availability of SARS-CoV-2 testing, the enhanced testing strategy begun on April 2 might have led to increased case detection among employees, compared with that among community members. Second, attack rates were calculated by department-group, shift, and compensation status; other characteristics that were not assessed might have contributed to risk for infection within the facility. Third, attack rates stratified by race and ethnicity are not reported because these data were incomplete. Fourth, unlike a recent study among meat processing employees (*7*), there was limited testing of asymptomatic persons in this study; therefore, the proportion of symptomatic infections reported here is likely an overrepresentation of the proportion of symptomatic SARS-CoV-2 infections in the population, and the number of cases identified is likely an underestimation of the number of SARS-CoV-2 infections in the population. Finally, the location of virus acquisition (e.g., facility versus community) for individual employees could not be determined.

These findings highlight the potential for rapid transmission of SARS-CoV-2 among employees in meat processing facilities. Employers should prioritize implementation of control measures consistent with published guidelines to mitigate the risk for occupational SARS-CoV-2 transmission (*1*,*2*). A robust mitigation program including engineering (e.g., modification of workstations to separate workers) and administrative (e.g., promoting social distancing when possible) controls should be implemented because no single control measure likely will eliminate transmission. Consistent and correct use of masks can prevent employees with COVID-19 from infecting others. Once a case is identified, prompt isolation of the infected employee and identification of contacts is necessary to reduce spread within the facility and the community. If widespread transmission continues despite these measures, temporary facility closure might reduce transmission among employees and their contacts.

**Summary**

**What is already known about this topic?**

Persons in congregate work settings are at increased risk for infection with respiratory pathogens, including SARS-CoV-2.

**What is added by this report?**

During March 16–April 25, 25.6% (929) of employees at a meat processing facility in South Dakota and 8.7% (210) of their contacts were diagnosed with COVID-19; two employees died. The highest attack rates occurred among employees who worked <6 feet (2 meters) from one another on the production line.

**What are the implications for public health practice?**

Implementing control measures before, or soon after, SARS-CoV-2 introduction into meat processing facilities, especially in areas where employees have prolonged, close contact with others, might substantially reduce the risk for SARS-CoV-2 spread within facilities.

## Footnotes

* Beginning April 12, the facility did not slaughter any more animals. During April 12–14, the facility processed animals that had already been slaughtered, shipped finished product, and progressively closed departments. From April 15 onward, only staff members necessary for maintenance, cleaning, and sanitization of the facility, transportation of remaining product, and implementation of COVID-19 prevention activities reported to work.

† Conversion is the process of further refining initial cuts of meat into finished fresh meat products.

§ U.S. Department of Health and Human Services, Title 45 Code of Federal Regulations 46, Protection of Human Subjects. https://www.ecfr.gov/cgi-bin/retrieveECFR?gp=&SID=83cd09e1c0f5c6937cd9d7513160fc3f&pitd=20180719&n=pt45.1.46&r=PART&ty=HTML.

¶ Public health departments can request access to the TIM system via this e-mail address: eocevent340@cdc.gov.
